# Extraction of Polyhydroxyalkanoates from Purple Non-Sulfur Bacteria by Non-Chlorinated Solvents

**DOI:** 10.3390/polym13234163

**Published:** 2021-11-28

**Authors:** Sara Filippi, Patrizia Cinelli, Andrea Mezzetta, Pietro Carlozzi, Maurizia Seggiani

**Affiliations:** 1 Department of Civil and Industrial Engineering, University of Pisa, L.go Lucio Lazzarino 1, 56122 Pisa, Italy; patrizia.cinelli@unipi.it (P.C.); maurizia.seggiani@unipi.it (M.S.); 2 Department of Pharmacy, University of Pisa, Via Bonanno, 6, 56126 Pisa, Italy; andrea.mezzetta@unipi.it; 3 Research Institute on Terrestrial Ecosystems, CNR, Via Madonna del Piano 10, 50019 Florence, Italy; pietromario.carlozzi@cnr.it

**Keywords:** purple non-sulfur photosynthetic bacteria, polyhydroxyalkanoates, extraction, non-chlorinated solvent, ionic liquids

## Abstract

In this study, non-chlorinated solvents such as cyclohexanone (CYC) and three ionic liquids, (ILs) (1-ethyl-3-methylimidazolium dimethylphosphate, [EMIM][DMP], 1-ethyl-3-methylimidazolium diethylphosphate, [EMIM][DEP] and 1-ethyl-3-methylimidazolium methylphosphite, [EMIM][MP]) were tested to extract polyhydroxyalkanoates (PHAs) from the purple non-sulfur photosynthetic bacterium (PNSB) *Rhodovulum* *sulfidophilum* DSM-1374. The photosynthetic bacterium was cultured in a new generation photobioreactor with 4 L of working volume using a lactate-rich medium. The extracted PHAs were characterized using a thermogravimetric analysis, differential scanning calorimetry, infrared spectroscopy, proton nuclear magnetic resonance and gel permeation chromatography. The most promising results were obtained with CYC at 125 °C with an extraction time of above 10 min, obtaining extraction yields higher than 95% and a highly pure poly(3-hydroxybutyrate-*co*-3-hydroxyvalerate) (PHB-HV) with around 2.7 mol% of hydroxylvalerate (HV). A similar yield and purity were obtained with chloroform (CHL) at 10 °C for 24 h, which was used as the referent solvent Although the three investigated ILs at 60 °C for 4 and 24 h with biomass/IL up to 1/30 (*w*/*w*) obtained PHAs strongly contaminated by cellular membrane residues, they were not completely solubilized by the investigated ILs.

## 1. Introduction

Polyhydroxyalkanoates (PHAs) are a very interesting class of biocompatible and biodegradable thermoplastic polyesters, mainly produced by prokaryotic organisms such as bacteria in response to various stress conditions and stored inside cells as carbon and energy reserves, in the form of water-insoluble granules (0.2–0.5 μm) [1]. Homo- or co-polyesters with different hydroxyl alkanoic acids can be obtained depending on the microorganisms used and cultivation conditions [2,3]. PHAs are completely biodegradable in several natural environments (industrial/home composting, soil, activated sludge, marine water) and do not form any toxic products [4].

The most common commercially available PHAs are poly-3-hydroxybutyrate (PHB) and poly-3-hydroxybutyrate-*co*-hydroxyvalerate (PHB-HV). The homopolymer PHB is a brittle material, while the HB-*co*-HV copolymer becomes more ductile with an increase HV content. Despite their potential, their extensive application is still limited due to their high cost (7–9 EUR·kg^−1^) [5,6] as compared to other bioplastics such as polylactate (PLA). For this reason, PHAs are mainly used in high-value applications as materials for drug delivery and medical device components [5,6]. The high production cost of PHA is a result of several factors, such as the use of pure or genetically modified cultures, the cost of the raw feedstocks used, such as substrates for the growth of bacteria, such as glucose, and the downstream processing for the recovery of PHA [5,6,7,8]. The latter significantly affects the process economics, accounting for up to 50% of the overall production cost and, also, its environmental sustainability [9]. In fact, the PHA extraction methods typically involve the use of halogenated solvents, such as chloroform (CHL), dichloromethane or 1,2-dichloroethane [10]. The listed solvents dissolve the lipid part of the non-PHA cell mass (NCPM), which can also easily be removed prior to PHA extraction by methanol or ethanol, and dissolve both short chain length (*scl*-) and medium chain length (*mcl*-) PHA [11], without dissolving any other NCPM components. After this, the PHA’s solubility is drastically reduced by adding a PHA anti-solvent, typically methanol, hexane, ether, acetone, or water. This leads to the precipitation of highly pure PHA. However, halogenated organic solvents, particularly CHL, have severe toxicity and, thus, a high environmental impact [12,13]. In fact, chloroform is suspected of causing cancer (i.e., possibly carcinogenic, IARC Group 2B), is classified as an extremely hazardous substance in the United States as defined in Section 3.2 of the U.S. Emergency Planning and Community Right-to-Know Act (42 U.S.C. 11002), and is subject to strict reporting requirements by facilities that produce, store, or use it in significant quantities. CHL is highly volatile (boiling temperature 61 °C and vapour pressure of 21.3 kPa at 20 °C) and has a Threshold Limit Value, TLV, of 10 ppm as its TWA (time-weighted average, that is, the time-weighted average concentration of the toxic substance over a normal 8 h workday and 40 h workweek, to which nearly all workers may be repeatedly exposed, every day, without adverse health effects) and 2 ppm as its TWA for skin contact. In addition, CHL is toxic to aquatic organisms. Consequently, less toxic non-halogenated solvents have been investigated to develop more environmentally friendly methods of solvent-based PHA extraction, such as acetic acid, acetic acid anhydride, n-methyl-pyrrolidone, tetrahydrofuran, acetic acid esters, γ-butyrolactone [14] and low-molecular ketones, such as acetone, methyl-isobutyl ketone, and cyclohexanone (CYC) [11]. The latter is not carcinogenic, but is moderately toxic, with a TLV of 25 ppm as its TWA and is not volatile (boiling temperature of 156 °C with vapour pressure of 0.67 kPa at 20 °C). Recently, Rosengart et al. [15] used anisole and CYC as solvents to extract PHB from *Burkholderia sacchari* cells, and obtained recovery yields of 97 and 93%, respectively, at 120–130 °C for 15–30 min with a cell/solvent ratio of 1.5% (*w*/*v*). CYC and γ-butyrolactone were used by Jiang et al. [14] to extract PHA from bacterial strain *Cupriavidus necator* H16, obtaining a recovery yield of 95% in the case of CYC after just 3 min at 120 °C, and of 50% with γ-butyrolactone after 60 min.

Another approach for PHA recovery relates to the digestion of the NCPM by chemicals such as sodium hypochlorite, cyclic carbonates, sodium hypochlorite or bio-catalysts (nucleases, phospholipases and lysozyme) with the consequent release of PHA granules [16,17,18]. With this approach, a neoteric class of green solvents, composed only by ions, ionic liquids (ILs), was used. Due to their excellent ability to dissolve the main biopolymers present in nature [19,20,21], some ILs have been used to remove the surrounding NCPM. PHA was extracted from a plant source using ILs, comprising cations such as ammonium, imidazolium, quinolinium, pyrazolium, oxazolum, isoquinolinium and anions as carboxylates, sulfosuccinates and sulfate [22]. Additionally, 1-Ethyl-3-methylimidazolium methylphosphonate [EMIM][MP] has been used to effectively disrupt cell walls of cyanobacteria extracting PHAs [23]. Dubey et al. [24] used 1-ethyl-3-methylimidazolium diethylphosphate ([EMIM][DEP]) to dissolve a wet bacterial biomass of *Halomonas hydrothermalis,* obtaining PHA extraction yield of 60% and purity of 86%. It is important to note that the toxicity of ILs is strongly affected by the type of cation and anion, and the interaction between the two, the alkyl chain length and the presence of functional groups [20,25]. Thus, it is not possible to make generalizations with regard to the toxicity of ILs, and each case must be studied in detail. In fact, ILs are widely used in life science applications, and as antimicrobial agents [26]. An application-specific selection of IL structure can assist in obtaining solvents with good performance and, in some cases, an adequate no-toxicity.

In this study, the purple non-sulfur photosynthetic bacterium (PNSB) *Rhodovulum* *sulfidophilum* DSM-1374 was cultured photoheterotrophically in a photobioreactor with 4 L of working volume, using a lactate-based medium in order to produce a PHA-containing biomass [27]. CYC was investigated for its use as a solvent of PHA, using different extraction temperatures and times to evaluate the optimal extraction conditions in terms of yield and extracted PHA purity.

In addition, on the basis of the possible combinations of ion pairs, by taking into account the hydrogen bonding receipt ability (β value) of the anion and hydrogen-bonding donating ability of cation (α value) [23], three ionic liquids (ILs) (1-ethyl-3-methylimidazolium dimethylphosphate, [EMIM][DMP], [EMIM][DEP] and 1-ethyl-3-methylimidazolium methylphosphite, [EMIM][MP]) were selected to extract PHA from the *Rhodovulum* *sulfidophilum* cells. These types of ionic liquids provided encouraging results, as previously described in the literature [23,24], and they have not been previously applied with PNSB. Chloroform was used as the reference solvent to assess the performance of the other investigated methods and solvents.

The recovered PHAs were characterized by thermogravimetric analysis (TGA), differential scanning calorimetry (DSC), Fourier-transform infrared (FTIR) spectroscopy, proton nuclear magnetic resonance (^1^H NMR) and gel permeation chromatography (GPC) and the results were compared with those of a commercial PHA sample.

## 2. Materials and Methods

### 2.1. Materials

CHL, CYC, 4-methyl-2-pentanone (MIBK), diethyl ether, 1-methylimidazole, 1-bromoethane, trimethyl phosphate, triethyl phosphate, dimethyl phosphite and methanol were supplied as A.C.S. reagents grade by Merck (Darmstadt, Germany) and used as received. A commercial sample of PHB-HV was supplied by NaturePlast (Ifs, France) under the trade name PHI 002^®^.

### 2.2. Bacterial Strain and Growth Conditions

The marine bacterium as *Rhodovulum sulfidophilum* DSM-1374 from the DSMZ collection, Germany, was grown photoheterotrophically [28] in a cylindrical photobioreactor (PBR) with 4 L of working volume, to produce PHA-rich biomass. The PBR was equipped with an internal shaped device with 4 paddles for mixing the culture that was operated indoors in batch growth conditions [29] under a 16/8 light/dark cycle. The synthetic-medium composition for culturing *Rhodovulum sulfidophilum* DSM-1374 was as follows (per 1.0 L): lactate (6.0 g), KH_2_PO_4_ (0.5 g), MgSO_4_ × 7H_2_O (0.4 g), NH_4_Cl (0.4 g), CaCl_2_ (0.05 g), yeast extract (0.3 g), 5 mL of Fe(III) citrate solution (0.1% in H_2_O), and 0.4 mL of vitamin B_12_ solution (10 mg in 100 mL H_2_O), and 1.0 mL of trace-element solution. The composition of trace-element solution (1.0 L) was as follows: ZnSO_4_ × 7H_2_O (0.1 g), MnCl_2_ × 4H_2_O (0.03 g), H_3_BO_3_ (0.3 g), CoCl_2_ × 6H_2_O (0.2 g), CuCl_2_ × 2H_2_O (0.01 g), NiCl_2_ × 6H_2_O (0.02 g) and Na_2_MoO_4_ × 2H_2_O (0.03 g) [27]. The medium composition was supplemented with 2% sodium chloride. The growth was carried out by using a phosphorus-free medium. The medium was sterilized by autoclaving for 20 min at 121 °C and at an absolute pressure of 2.0 atm. Two lamps placed opposite one another (OSRAM power-star HQI-TS lamps, 150-W each) illuminated the external surface of the PBR with an average irradiance of 80.8 ± 4.5 W/m^2^. A pH-probe and a termoresistance PT100 were inserted into the cultural broth and connected with a unit for the automatic control of pH and temperature, respectively. A magnetic stirrer, positioned under the bottom section of the PBR, was used to mix the culture. The initial pH of the culture was 8.0, after which it was automatically controlled by adding the HCl solution (10 mM), using an external pump. The culture temperature was maintained at 30 ± 0.5 °C, using an external refrigerated-heating circulator (Julabo, model F25-HL, Seelbach, Germany) equipped with a heating/cooling finger immerged into the culture broth [29]. The production was carried out for 120 h after which the bacterial biomass was harvested, recovered by centrifugation, after which the cells were washed twice with a physiological solution, and were, subsequently, lyophilized for the characterization and extraction steps.

### 2.3. Analytical Methods

The cell dry weight (CDW) was determined by sampling 5 mL of culture that was diluted to 50 mL with distilled water, which was filtered (without compact cells) through a pre-weighed membrane with a 0.45 μm pore size (Sartorius GmbH, 3400 Göttingen, Germany). The cells were suspended again in 50 mL of distilled water, filtered and dried at 105 °C until a constant weight was reached [30]. The lactic acid concentration was determined by High-Performance Liquid Chromatography (HPLC) and Thermo Finnigan-Spectra System 6000 L P (San José, CA, USA); an aqueous solution of 0.1 wt.% H_3_PO_4_ was used as the mobile phase at a flow rate of 1.0 mL/min [31,32]. The concentration of poly-3-hydroxybutyrate (PHB) in *Rhodovulum sulfidophilum* culture was checked against the above-mentioned HPLC using a Synergy-Hydro-RP C-18 column (250 × 4.6 mm^2^ id). A mobile phase comprising 15 % (*v*/*v*) acetonitrile in 0.1 % (*v*/*v*) H_3_PO_4,_ in an aqueous solution, was employed at a flow rate of 1 mL/min. A commercial PHB (Biomer, Krailling, Germany) was used as the standard. Samples were taken daily (at the beginning and at the end of light period) from the PBR and placed into a pre-weighed tube, and centrifuged at 9000 rpm for 10 min. The pellet was frozen at −20 °C for acid digestion to crotonic acid by boiling the sample in 1 mL of pure sulfuric acid in screw-cap glass test tubes for 30 min. The extracts were diluted with water, filtered and injected into the HPLC for analysis [28]. The compound was detected at 214 nm. The analyses of bacterial dry-biomasses were performed using approximatively 6.0 mg of *Rhodovulum* dry-biomass, and further details have been reported elsewhere [28]. The average productivity of PHB (*PHB-P_avg_*) (mg/L day) was determined by *t* Equation (1), as follows:

(1) $$PHB-P_{avg}=\frac{PHB_{f}-PHB_{i}}{\left(t_{1}-t_{0}\right)}\;$$

where *PHB_f_* and *PHB_i_* are, respectively, the final and the initial concentrations of *PHB* in the cultures (mg/L) and *t*_1_ and *t*_0_ are the respective times.

The irradiance was measured with a Quantum/Radiometer/Photometer (model LI-250A, LICOR, Lincoln, NE, USA) equipped with a Pyranometer sensor, model number LI-200.

### 2.4. Synthesis of Ionic Liquids

The precursor 1-ethyl-4-methylimidazolium bromide ([EMIM]Br) was obtained following the procedure previously reported for bromide ionic liquids [33]. Both 1-bromoethane (110 mmol, 1.2 equiv) and 10 mL of 4-methyl-2-pentanone (MIBK) were added to a three-necked flask. A solution of 1-methyllimidazole (122 mmol, 1 equiv) in MIBK (20 mL) was added dropwise under magnetic stirring at room temperature, and the solution was stirred for 15 min. The reaction mixture was then heated to 80 °C and stirred for 12 h. Next, 25 mL of diethyl ether was added to the resultant mixture and the obtained solid was filtrated under vacuum, washed with MIBK and dried under vacuum to produce white solids at excellent yields.

The [EMIM][DMP], [EMIM][DEP] and [EMIM][MP] were synthetized using a previously reported procedure [34]. The correct amount (36 mmol, 1.2 equiv) of alkyl phosphate and of alkyl phosphite (trimethyl phosphate, triethyl phosphate and dimethyl phosphite) was added to [EMIM]Br (30 mmol, 1 equiv), respectively. The solution was heated at 110 °C and stirred for 24 h under argon. Then, the reaction mixture was repeatedly cooled down and washed with diethyl ether to remove the excess reagent. The recovered liquid was dried under vacuum for 2–3 h at 80 °C.

### 2.5. PHA Extraction with Chloroform

The lyophilized biomass was first extracted using CHL, which was chosen as the standard solvent to evaluate the maximum extractable PHA content in the investigated PNSB.

Around 1.0 g of biomass was suspended in 30 mL of CHL, and the mixture was gently stirred for 24 h at 10 °C. The treatment time and temperature were selected in order to optimize the extraction time and yield, preserving the PHA chain length. The protocol used was an intermediate between that used by Ramsey et al. [35], of 25 °C for 12 h, and Rosengart [36], of 4 °C for 36 h. The treatment was able to dissolve PHA and the cell membrane was not solubilized. The suspension was filtered under vacuum, using a Sartorius apparatus equipped with a Teflon filter (0.45 mm). Then, 120 mL of methanol was added to the filtrate to precipitate PHA and the resultant solution was stirred for 5 min. The solid was recovered by filtration, washed with fresh methanol (about 10 mL), and dried at 30 °C under vacuum at pressure of 10 mbar to remove CHL and methanol. The resultant solid was weighed and subjected to subsequent characterizations.

### 2.6. PHA Extraction with Cyclohexanone

Using two different extraction temperatures, 100 and 125 °C, and different extraction times: 5, 10, 20 and 30 min, 1.5 g of lyophilized biomass was treated with 25 mL of CYC, to evaluate their effect on the process kinetics and yield. After extraction, the mixture was filtered in vacuum to separate the membrane debris. Following this, 100 mL of methanol was added to the filtrate and the resultant solution was stirred for 5 min to complete the precipitation of PHA. The solid was recovered by filtration, washed with fresh methanol (around 30 mL) and dried at 80 °C under vacuum at a pressure of 10 mbar to remove CYC and methanol. The resultant solid was weighed and subjected to subsequent characterizations.

### 2.7. PHA Extraction with Ionic Liquids

The treatment with the three investigated ILs was conducted at higher biomass/IL ratios, 1/10 and 1/30 (*w*/*w*), the mixture was stirred at 60 °C from 4 to 24 h. The mixture was centrifuged at 4000 rpm for 10 min to recover the insoluble PHA, given the viscosity of the ILs. In fact, the viscosities at 60 °C of the used ILs are 58, 50 and 23 cP for [EMIM][DEP] [37], [EMIM][DMP] [38] and [EMIM][MP] [23,39], respectively.

The supernatant was removed and the solid was washed with methanol four times (5 mL per wash). Washing was carried out by the resuspension of the solid in methanol and the subsequent centrifugation and removal of the supernatant. Finally, the resultant solid was placed in an oven at 60 °C under vacuum for 12 h, weighed and subjected to subsequent characterizations.

### 2.8. Extracted PHA Characterization

The extracted PHAs were characterized using different analyses. Proton Nuclear Magnetic Resonance (^1^H-NMR) spectroscopy was carried by a Bruker-400 UltraShield, 400 MHz, using CDCl_3_ as the solvent in order to evaluate the type of PHA. Chemical shifts were referred to CDCl_3_ (δ = 7.25 ppm).

Fourier-Transform Infrared (FTIR) spectroscopy was conducted in ATR (Attenuated Total Reflectance) mode, using a Perkin Elmer Spectrum One spectrometer (Milano, Italy), equipped with a ZnSe crystal ATR device.

A thermogravimetric analysis (TGA) was performed on a TA Instruments TQ-500 (Milano, Italy), using 10–15 mg of sample in platinum pans, under a nitrogen atmosphere, from 30 to 800 °C with a heating rate of 10 °C/min.

Differential scanning calorimetry (DSC) was conducted on a Perkin Elmer Pyris I (Milano, Italy), using 10–15 mg of sample closed in aluminium pans. The samples were subjected to an initial heating from 50 to 200 °C, with a heating rate of 10 °C/min and were left at 200 °C for 5 min so as to destroy the crystallites and cancel the sample thermal history. Then, they were cooled to 50 °C with a cooling rate of 10 °C/min and, finally, subjected to secondary heating from 50 to 200 °C, with a heating rate of 10 °C/min.

Gel permeation chromatography (GPC), used to evaluate M_n_ (number average molecular weight), M_w_ (weight average molecular weight) and dispersity (M_n_/M_w_) of the PHA samples, was carried out by dissolving the sample in chloroform (4 mg/mL) and using a Jasco (Cremella, Italy) Gel Permeation Chromatography apparatus, equipped with a PU-2089 Plus pump, two detectors, a RI-4030 and a UV-4075, a Peltier column oven CO-4060 and an AS-2055 plus autosampler. The tests were performed at 25 °C using a Phenogel column, of 5 µm, pore size 10^4^ Å, 300 × 7.8 mm^2^ from Phenomenex (M_w_ operating range from 5000 to 500,000 Da). The volume of the injected solution was 50 µL. The flow rate of the solvent was 1.0 mL/min. Samples were filtered before injection, using a 0.45 µm PTFE filter. The instrument was calibrated using monodispersed polystyrene samples, tested in the same conditions, to correlate retention times to molecular weights.

To evaluate the filmability of the extracted PHA samples, bioplastic films were prepared using the solvent casting method. The recovered PHA sample (150 mg) was dissolved in 20 mL of CHL at room temperature. The solution was poured in a Petri dish with a diameter of 80 mm. The solvent was gradually evaporated at room temperature for 48 h and finally at room temperature under vacuum pressure for 2 h.

## 3. Results and Discussion

### 3.1. Production of PHA-Containing Bacterial Biomass

**Figure 1:**
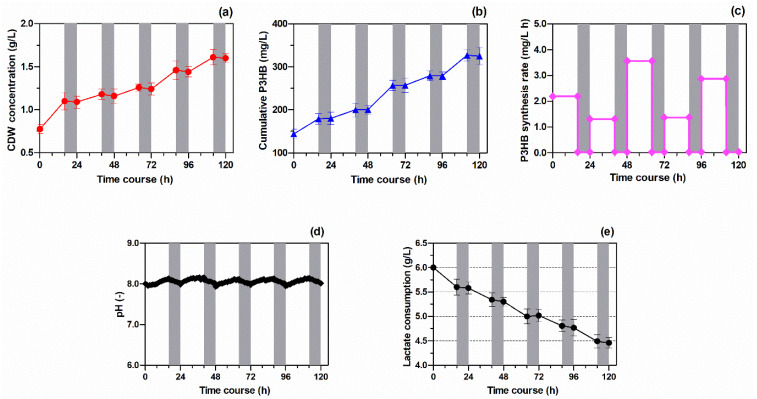
(**a**) CDW concentration; (**b**) Cumulative PHB in *Rhodovulum sulfidophilum* culture during batch growth conditions and 16/8 light/dark cycle. Grey bars represent dark periods. Data are mean ± SE; (**c**) PHB synthesis rate culture during 16/8 light/dark cycle; (**d**) pH trend and (**e**) lactate consumption. Grey bars represent dark periods.

Over 120 h of bacterial growth, the CDW concentration increased by up to 1.61 g/L. During that time, a notable accumulation of PHB in the *Rhodovulum sulfidophilum* culture was observed during the light time. As shown in Figure 1a,b, no growth and no PHB synthesis were observed in the dark, showing that the process is light dependent in *Rhodovulum sulfidophilum* DSM-1374. The highest concentration of PHB was 325 mg/L and the average PHB-productivity (PHB-P_avg_) was 36.2 mg/L day. Over 120 h of biomass growth, the polyester synthesis rate changed day by day and the maximum value (3.56 mg/(L·h)) was observed in light, during forty-eighth to sixty-eighth hours, as shown in Figure 1c. The trend of culture pH changed over time, as shown in Figure 1d. The pH value increased significantly during the light periods and decreased during dark periods. During bacterial growth, the lactate carbon source was consumed, for which the results are shown in Figure 1e. Moreover, it can be noted that the lactate consumption, which occurred only under light conditions, was inversely proportional to the bacterial growth; the higher the CDW concentration, the lower the quantitative of lactate in the *Rhodovulum* culture (Figure 1a). The average content of PHB in the dry-biomass of *Rhodovulum sulfidophilum* at the end of the experiments was 14.2 wt.%.

It is known that specific growth strategies are required in bacteria to accumulate PHAs, such as an excess supply of carbon and a limitation of nitrogen (N), phosphorus (P), sulfur (S) or magnesium (Mg) [29,32,40]. In this study, the excess supply of carbon (lactate 6.0 g/L), under P-starvation conditions, was proposed to enhance polymer production. Golomysova et al. [41] reported P3HB and polysaccharide polymer accumulation, during active bacterial growth, after feeding with organic acids (OAs). Metabolic pathways for the production of PHAs from the selected carbon source have been also reported by McKinlay and Harwood [42] and McCully and McKinlay [43]. PHA production by means of marine purple bacteria cultured in OAs-rich media was recently reported [29]. The authors demonstrated that *Rhodovulum sulfidophilum* DSM-1374 grown in a medium P-limited, containing lactate, increased the PHA concentration in *Rhodovulum* culture to 1000 mg/L, when grown using a semi-continuous regimen, under continuous illumination.

When a different PNSBs, such as *Rhodopseudomonas* sp. S16-VOGS3, were cultured (outdoors) in the 70-L tubular photobioreactor, the PHB concentration reached 377 mg(PHB)/L in the culture [44]. In this study, the highest concentration of PHB was found to be lower (325 mg/L culture). The comparison between the average PHB productivity (PHB-P_avg_) demonstrated that outdoors, the PHB-P_avg_ was 63 mg/L day and reduced to 36.2 mg/L day in the present investigation. The significantly different results described above could be attributed to several aspects such as: (1) the very different light conditions that were high outdoors and low indoors; (2) the N-limitation nutrient outdoors against the P-limitation nutrient indoors and (3) the different genera of the microorganisms utilized.

### 3.2. PHA Extraction with Chloroform and Cyclohexanone

**Figure 2:**
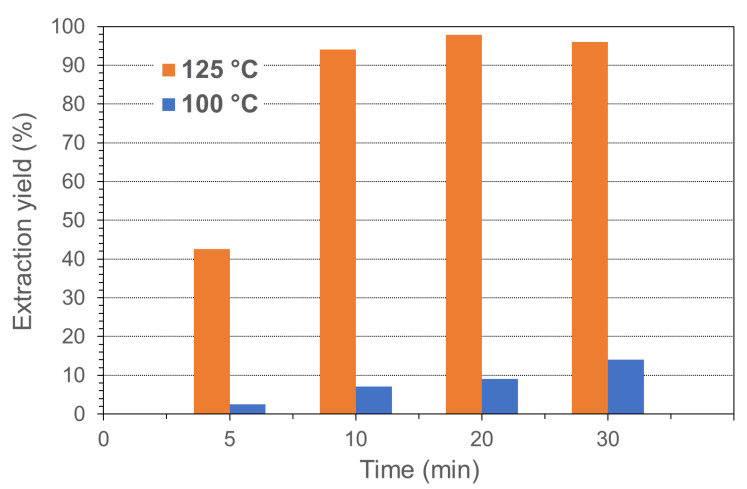
Extraction yields using CYC as function of temperature and time.

The extraction conducted on the lyophilized biomass using CHL at 10 °C for 24 h produced, after precipitation with methanol and drying, a residual solid corresponding to 14.0 wt.% of the initial dry biomass. This value is in accordance with the average content of PHB assessed in the dry-biomass, showing an almost complete extraction and recovery of PHA. The extraction yield for the extraction carried out with CYC was strongly influenced by the temperature, and yields higher than 95% were reached at 125 °C with extraction time above 10 min, as shown in Figure 2.

These results are in agreement with those obtained in the literature [14,15,45]. However, it is important to highlight that the extraction yield, with a specific solvent, depends on multiple factors, such as, (i) the bacterial strain and, consequently, the cell membrane that may have different permeability with the extraction solvent; (ii) the PHA content; (iii) the bacteria/solvent weight/volume % ratio; (iv) the extraction temperature and (v) the extraction time. In Table 1, the results we obtained with CYC and *Rhodovulum* *sulfidophilum* DSM-1374 (first line) are compared with those of other research groups that used the same solvent but different bacterial strains and conditions.

Jiang et al. [14] extracted PHA produced by the bacterial strain *Cupriavidus necator* H16, cultivated on vegetable oil as a sole carbon source, which has a PHA content of 82.3 wt.%. Its treatment at 120 °C for 3 min, with a bacteria/CYC ratio of 2.0% g/mL, allowed for the recovery of 99% of the PHB from the biomass with a similar purity to that extracted using chloroform. Rosengart et al. [15] extracted PHA produced by the bacterial strain *Burkholderia sacchari* DSM 17165, grown on different types of simple sugars, with a PHA content of 57.5 wt.%. The extraction yield was 98% at 120–130 °C for 15 min, using a bacteria/CYC ratio of 1.5% g/mL. When the ratio was increased to 6.0% g/mL, which was the case for this study, the yield decreased to 89%, even after 30 min of treatment. Finally, Van Walsem et al. [45], recovered the 80% of PHA in *Escherichia coli*, having a PHA content of 80.0 wt%, working at 120 °C for 3 min, with a bacteria/CYC ratio of 8.0% g/mL.

**Figure 3:**
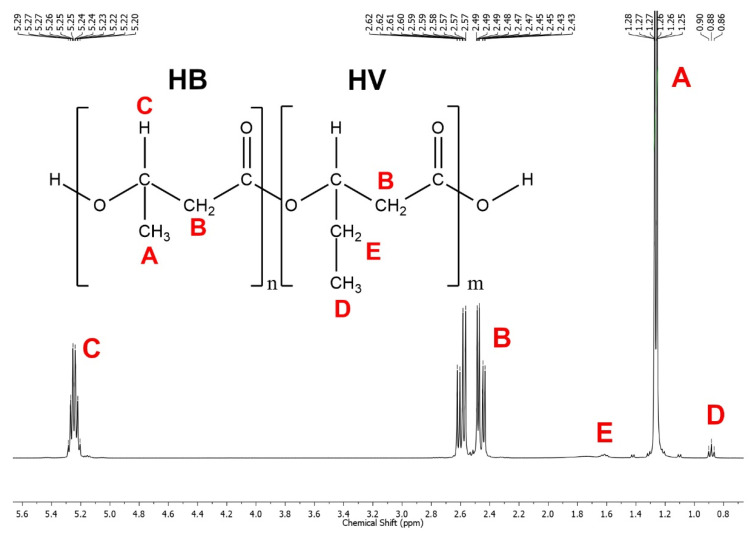
Correspondences between the ^1^HNMR signals of PHA extracted using CHL at 10 °C for 24 h and the protons of the polymer structure. The letters A, B etc. indicate the signals due to protons having the same letter in the chemical structure.

In Figure 3, as an example, the correspondences between the different NMR signals and the protons of the polymer extracted using CHL are indicated. ^1^H NMR spectra of PHAs, extracted by the CYC and commercial PHB-HV sample were found to be very similar to that extracted by CHL, showing that both solvents (CHL and CYC) led to the recovery of high purity PHAs. An ^1^HNMR analysis also showed that the extracted PHAs correspond to poly(3-hydroxybutyrate-*co*-3-hydroxyvalerate), PHB-HV. In fact, the spectra results were comparable to that of the commercial PHB-HV (PHI002 by NaturePlast). According to the literature [46], using Equation (2), hydroxyvalerate (HV) molar contents of 0.9, 2.2 and 2.7 mol% were calculated for the commercial PHB-HV and for the PHA extracted by CHL and CYC, respectively.

(2) $$HV\;\left(\%\right)=\frac{Area\;CH_{3}\left(HV\right)}{Area\;CH_{3}\left(HV\right)+Area\;CH_{3}\left(HB\right)}\times 100$$

**Figure 4:**
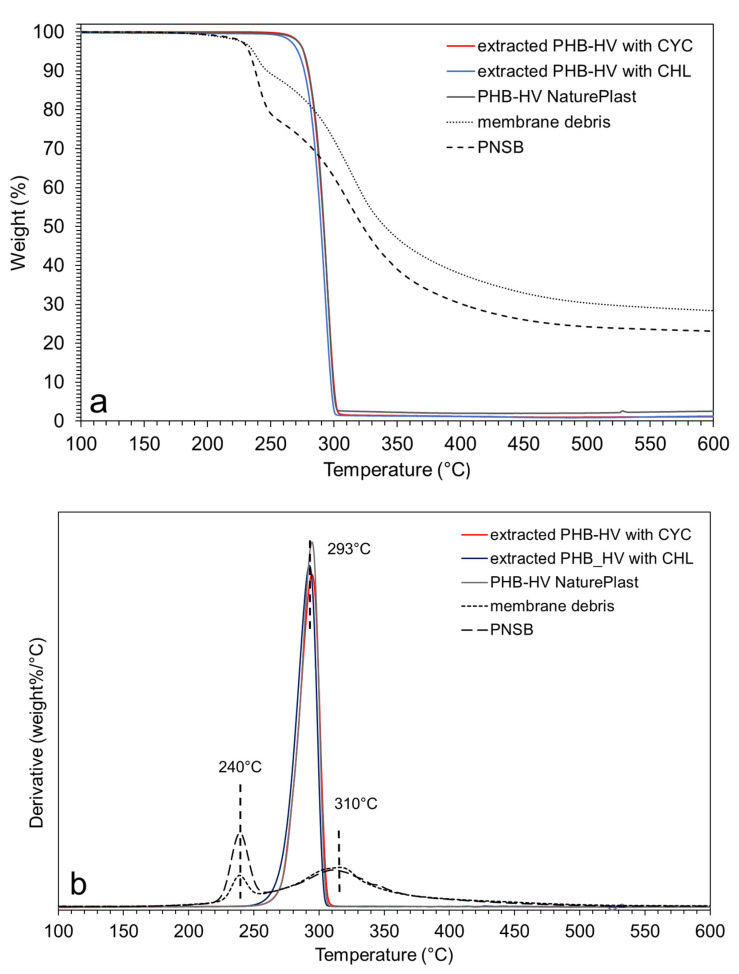
(**a**) TG and (**b**) DTG curves of PHB-HV extracted by CHL, CYC (125 °C, 20 min), commercial PHB-HV, purple bacteria and membrane debris after extraction by CYC.

The TG (thermogravimetric) and DTG (derivative thermogravimetric) curves of extracted PHB-HVs are shown in Figure 4a,b together with those of the commercial PHB-HV sample, freeze-dried bacterial biomass before the extraction of the PHA content (PNSB) and membrane debris after extraction using CYC. Both PHB-HVs show a single main event of thermal decomposition with a peak at 294 and 296 °C for the samples extracted by CHL and CYC, respectively. The residues at 600 °C were negligible for the extracted PHB-HVs and for the commercial PHB-HV, confirming their high purity. The thermal stability of the copolymer extracted using CHL seems slightly lower than that of the copolymer extracted by CYC that is very similar to that of the commercial sample.

Therefore, even if the CHL treatment had been carried out at a low temperature, the chlorinated solvent would have caused a slight polymer degradation. According to Ramsay et al. [35], degradation by CHL occurs for temperatures above 100 °C and refers to depolymerization (reduction of MWs). The extraction temperature was low, but the duration was quite long (24 h instead 12 h). Moreover, the bacterial biomass was different from that of [35] and the cellular membrane could be attacked by a solvent with different kinetics. Further investigation is required to define the causes of this slight degradation. The DTG curve of the bacterium shows two main decomposition events with a first peak at 240 °C and for the second, in a wider range of temperatures, with a peak at around 310 °C. These results suggest that the decomposition temperature of PHB-HV inside the bacterial membrane seems to occur at a lower temperature than that of the extracted polymer. Thus, the cell membrane seems to have an accelerating effect on the pyrolysis of the biopolymer, which decomposes in the same temperature range of the first pyrolysis event of the membrane components. It should also be noted that the extraction process would be similar. In fact, the PHB-HV extracted is equal to 13.9% of the biomass and the residue of pyrolysis of PHB-HV at 600 °C is negligible, and from the pyrolysis residue at 600 °C of bacterial biomass (24%), it is possible to estimate the percentage residue due to the non-PHA cell mass (NCPM) alone, which corresponds to 28%, which is in agreement with the experimental data (29%) (Figure 4).

**Figure 5:**
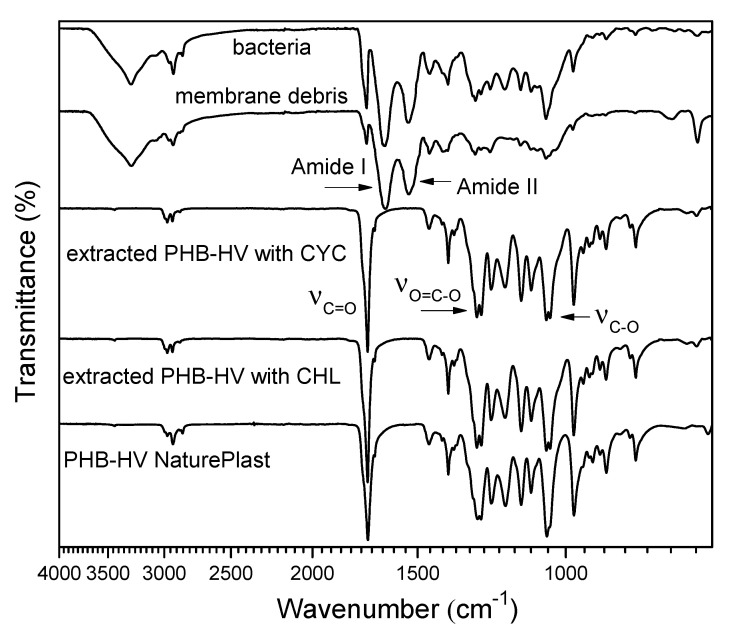
FTIR analysis of PHB-HV extracted with CHL, CYC (125 °C, 20 min), commercial PHB-HV, starting purple bacteria and membrane debris after extraction with CYC.

The FTIR spectra, reported in Figure 5, confirmed the high purity of the extracted PHB-HVs with CHL and CYC, demonstrating a correspondence between all the signals with those of the commercial sample. In fact, the spectra of both of the extracted PHB-HV samples show the characteristics signals of the ester functionality as the stretching C=O vibration at 1720 cm^−1^ and the stretching C-O vibration around 1270 and 1050 cm^−1^, and they do not show any of the most important signals related to the cell membrane, due to protein chains. The two prominent bands at 1645 and 1545 cm^−1^, usually referred to Amide I and Amide II bands [47], corresponded to C=O stretching vibrations of the peptide bond and the C-N stretching vibrations and N-H bending modes, respectively, but are completely absent in the PHB-HV patterns.

**Figure 6:**
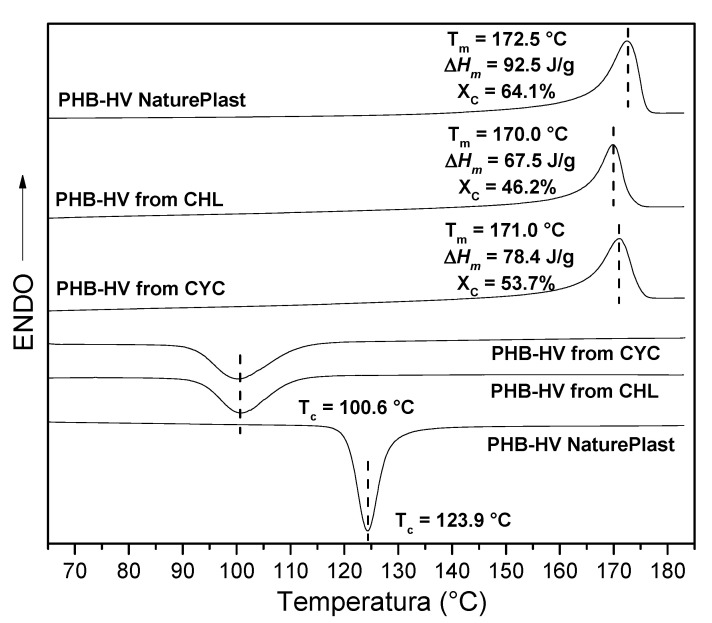
2nd heating and cooling DSC curves and derived calorimetric data of PHB-HV extracted with CYC (125 °C, 20 min), extracted with CHL (10 °C, 24 h) and commercial PHB-HV.

The DSC curves of the extracted PHB-HVs and commercial PHB-HV are reported in Figure 6, together with the derived calorimetric data. The crystallinity X_C_, expressed in percentage, was calculated using the ratio between the melting enthalpy of the sample, Δ*H*_m_. The calculated melting enthalpy of pure PHB crystals corresponded to 146 J/g [48]. As shown, the PHB-HVs, extracted using CHL or CYC, show similar thermal properties with crystallinity of 46.2 and 53.7%, respectively. The crystallization process is kinetically slower than that of commercial sample as shown by the lower T_c_ (100.6 °C) with respect to 124 °C of the commercial sample. These data are perfectly in accordance with the higher HV content (2.2 and 2.7 mol%) of the extracted PHB-HVs compared to that of the commercial sample (0.9 mol%). It is known that the minor HV comonomer units are excluded in the crystal lattice of the major HB comonomer units, and this hinders the crystallization process, resulting in a decreased degree of crystallinity and a lower crystallization temperature [48].

**Figure 7:**
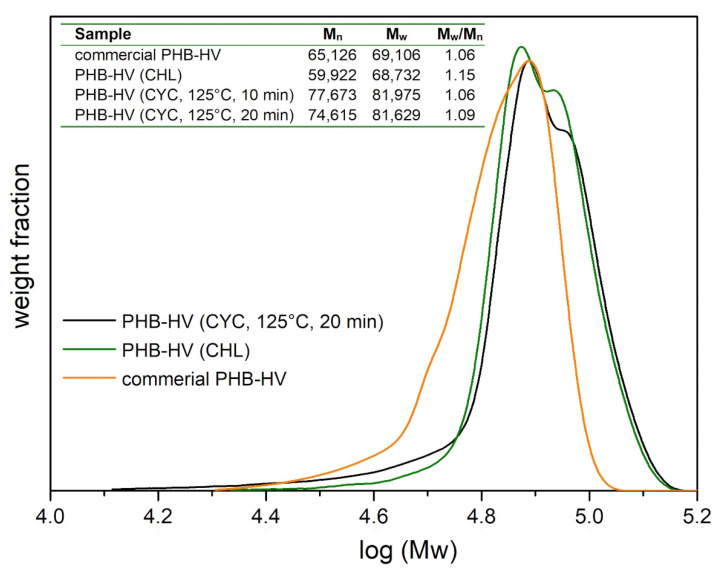
GPC results for PHB-HV extracted with CHL and CYC.

To conclude, the results of the GPC analysis are reported in Figure 7. The weight molecular distributions for the two extracted PHB-HVs are similar, with higher values of M_n_ and M_w_ (expressed in g mol^−1^) for the PHB-HV extracted by CYC. This confirms that the CYC-based extraction procedure, even at high temperatures (125 °C), did not adversely affect the average polymer chain length compared to the CHL-based procedure at low temperatures (10 °C). On the contrary, the procedure with CYC led to the recovery of a PHB-HV, with the same yield, producing a higher crystallinity and longer chain length. In addition, an increase in the extraction time, from 10 to 30 min, had no significant effect on M_n_ and M_w_.

Finally, in order to qualitatively evaluate the filmability by casting of the recovered biopolymers, bioplastic films were prepared, using the solvent casting method. The recovered PHB-HVs were dissolved in chloroform and poured into a glass Petri dish. The solvent was evaporated at room temperature and a film was obtained after evaporation. The digital images of the starting recovered wet PHB-HV, extracted by CYC, and the corresponding film obtained by casting are showed in the graphical abstract. Films with the same consistency and opacity were obtained using PHB-HV obtained by CHL extraction and commercial PHB-HV.

### 3.3. PHA Extraction with Ionic Liquids

PHA extraction from a bacteria biomass using ILs has been the subject of a Patent [22] and some studies. Kobayashi et al. [23] proposed a recovery process for PHA accumulated in cyanobacteria (with PHA content of 5 wt.%), using [EMIM][DMP], recovering the insoluble PHA by filtration. They observed that the solution viscosity became too high with a bacteria concentration of higher than 0.1 wt.% and difficult filtration. For this reason, they used 1.0 g of ILs for 1.0 mg of dried cyanobacteria, which are not concentrated enough to be suitable for industrial applications. Despite the use of these extreme conditions, the recovered PHB was contaminated by membrane residues and the PHB content resulted in around 30 wt.% [23].

**Figure 8:**
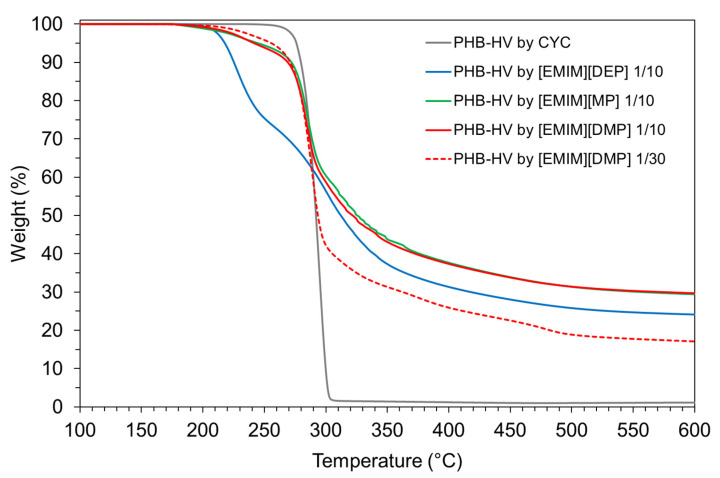
TG curves of PHB-HV extracted with ILs for 24 h at 60 °C compared with the sample extracted by CYC.

In this study, the experiments were conducted at higher biomass/IL ratios, 1/10 and 1/30 (*w*/*w*). Initially, a weight biomass/IL ratio of 1/10 and an extraction time of 4 h at 60 °C was used for all three investigated ILs. Under these conditions, it was possible to recover the insoluble PHA fraction by centrifugation using only [EMIM][DEP]. Furthermore, while using [EMIM][DMP] and [EMIM][MP], the PHA insoluble fraction did not separate from the solution. In order to induce PHA coagulation, a few drops of methanol were added as antisolvent. It was difficult to recover coagulated biopolymer by filtration due to the high solution viscosities, also evidenced by Kobayashi et al. [23] The wet filtrated solid was washed with methanol and the amount of dried solid residues obtained for 1 g of treated biomass resulted in 0.45, 0.69 and 0.72 g, using [EMIM][MP], [EMIM][DMP] and [EMIM][DEP], respectively. These values show that the amount of recovered solid after IL treatment was well above the quantity of PHB-HV present in the starting biomass (14.3%), showing a relevant presence of NPCM. On the basis of these results, further tests were conducted for which the treatment time was increased (from 4 to 24 h) and the biomass/ionic liquid *w*/*w* ratio was reduced, from 1/10 to 1/30. However, in all these tests, the recovered biopolymer resulted in contamination by a high amount of membrane debris. TG curves of dried recovered solids after 24 h of treatment with the three ILs with a biomass/IL ratio of 1/10 (*w*/*w*), using [EMIM][DMP] with biomass/IL ratio of 1/30 (*w*/*w*) are compared with the PHB-HV extracted by CYC in Figure 8. As shown, all the extracted samples are heavily contaminated by NPCM, with a PHB-HV content of around 30% (evaluated as weight loss in the temperature range of PHB-HV decomposition). This content increases to around 50% with more dilute conditions, as shown with [EMIM][DMP] (Figure 8). The TG curves of recovered solids are similar to those obtained by Dubey et al. [24] using [EMIM][DMP] to extract PHA from *Halomonas hydrothermalis* bacteria (with a PHA content of 74 wt.%) with a weight ratio biomass/IL of 1/10 and methanol to recover PHA by precipitation as performed initially in this work. They obtained PHB with a 60% yield and a purity of 86%, determined by the N content, indicating the presence of amino acid impurities in the recovered biomass.

**Figure 9:**
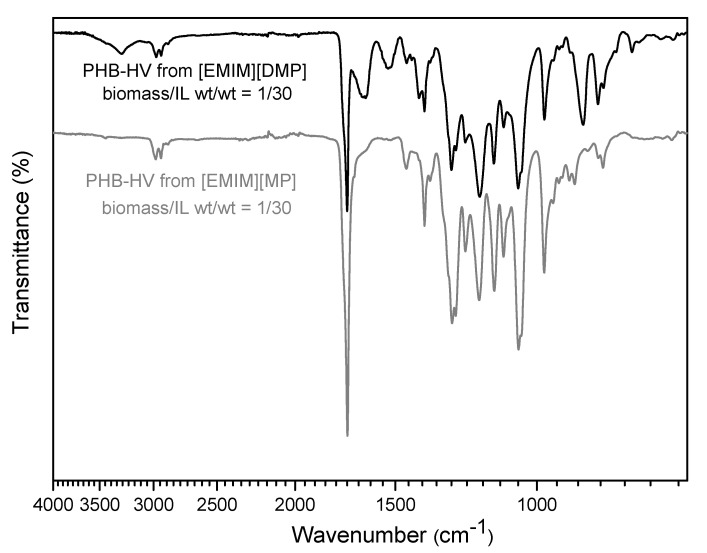
FTIR spectra of PHB-HV from [EMIM][DMP] and [EMIM][MP].

The FTIR spectra of the extracted solids with [EMIM][MP] and [EMIM][DMP] in diluted conditions after 24 h, reported in Figure 9, show a good purity using [EMIM][MP] under these conditions.

Given the significant contamination of PHB-HV by residual cellular material, further characterizations were not carried out on these samples. Further tests are needed to obtain higher NPCM removal efficiencies and, consequently, higher PHB-HV purity to make the procedure with ILs applicable on industrial scale.

## 4. Conclusions

In this work, *Rhodovulum sulfidophilum* DSM-1374 was cultured using a 16/8 light-dark cycle, obtaining a PHB content of 14.3% in the dry biomass. The excess supply of carbon (lactate 6.0 g/L) and P-starvation conditions was proposed to enhance polymer production. CYC and three ionic liquids ([EMIM][DEP], [EMIM][MP] and [EMIM][DMP]) were investigated to extract PHA from bacterial biomass, as alternatives to commonly used halogenated solvents such as CHL. Recovery yields higher than 95%. were obtained using CYC at 125 °C, after 10 min. The extracted PHA was confirmed to be PHB-HV with 2.7 mol% of HV, with a purity, thermal properties, molar mass and dispersity similar to those of the PHB-HV extracted with CHL, which was used as the reference solvent, and of a commercial PHB-HV. These results showed that CYC at 125 °C presents a valuable alternative to CHL in the extraction of PHAs from bacterial biomasses with the requirement of only limited extraction times (10–20 min).

On the contrary, the three investigated ILs obtained PHAs highly contaminated by cellular membrane residues. Using [EMIM][MP] with a biomass/IL ratio of 1/30 (*w*/*w*) after 24 h, the extracted PHB-HV showed a good purity. Consequently, further investigations are required regarding the use of ILs as PHA extraction solvents, to increase the selectivity of extraction and overcome the difficulties that occur during the filtration/centrifugation/washing steps, because of their high viscosity.

## Figures and Tables

**Table 1:** PHA extraction with CYC. A comparison with literature data.

Paper	Bacterium Strain	PHA Content (wt.%)	bacteria/CYC Ratio wt/v % (% g/mL)	Extraction Temperature (°C)	Extraction Time (min)	Extraction Yield (%)
this one	Rhodovulum Sulfidophilum DSM-1374	14.0	6.0	125	5	43
			6.0	125	10	95
			6.0	125	20	98
[14]	Cupriavidus necator H16	82.3	2.0	80	1200	16
			2.0	100	5	90
			2.0	120	3	99
[15]	Burkholderia sacchari DSM 17165	57.7	1.5	120–130	15	98
			6.0	120–130	15	87
			6.0	120–130	30	89
[45]	Esch. coli	80.0	7.0	90	35	80

## Data Availability

The data presented in this study are available on request from the corresponding author.

## References

[1] Sudesh K., Abe H., Doi Y. (2000). Synthesis, Structure and Properties of Polyhydroxyalkanoates: Biological Polyesters. Prog. Polym. Sci..

[2] Muthuraj R., Valerio O., Mekonnen T.H. (2021). Recent Developments in Short- and Medium-Chain- Length Polyhydroxyalkanoates: Production, Properties, and Applications. Int. J. Biol. Macromol..

[3] Yashavanth P.R., Meenakshi D., Soumen K.M. (2021). Recent Progress and Challenges in Cyanobacterial Autotrophic Production of Polyhydroxybutyrate (PHB), a Bioplastic. J. Environ. Chem. Eng..

[4] Tokiwa Y., Calabia B., Ugwu C., Aiba S. (2009). Biodegradability of Plastics. Int. J. Mol. Sci..

[5] Sabapathy P.C., Devaraj S., Meixner K., Anburajan P., Kathirvel P., Ravikumar Y., Zabed H.M., Qi X. (2020). Recent Developments in Polyhydroxyalkanoates (PHAs) Production—A Review. Bioresour. Technol..

[6] Bugnicourt E., Cinelli P., Lazzeri A., Alvarez V. (2014). Polyhydroxyalkanoate (PHA): Review of Synthesis, Characteristics, Processing and Potential Applications in Packaging. Express Polym. Lett..

[7] Salehizadeh H., Van Loosdrecht M.C.M. (2004). Production of Polyhydroxyalkanoates by Mixed Culture: Recent Trends and Biotechnological Importance. Biotechnol. Adv..

[8] Pérez-Rivero C., López-Gómez J.P., Roy I. (2019). A Sustainable Approach for the Downstream Processing of Bacterial Polyhydroxyalkanoates: State-of-the-Art and Latest Developments. Biochem. Eng. J..

[9] Narodoslawsky M. (2015). LCA of PHA Production—Identifying the Ecological Potential of Bio-Plastic. Chem. Biochem. Eng. Q..

[10] Holmes P.A., Wright L.F., Alderson B., Senior P.J. (1980). A Process for the Extraction of Poly-3-Hydroxy-Butyric Acid from Microbial Cells European Patant. No. EP0015123A1. https://patentimages.storage.googleapis.com/4d/68/b0/ef3bd1590af616/EP0015123A1.pdf.

[11] Koller M., Niebelschütz H., Braunegg G. (2013). Strategies for Recovery and Purification of Poly[(R)-3-Hydroxyalkanoates] (PHA) Biopolyesters from Surrounding Biomass. Eng. Life Sci..

[12] Information Resouces Management Association (2017). Public Health and Welfare: Concepts, Methodologies, Tools, and Applications.

[13] IARC, International Agency for Research on Cancer (1999). Monographs on the Evaluation of Carcinogenic Risks to Humans.

[14] Jiang G., Johnston B., Townrow D., Radecka I., Koller M., Chaber P., Adamus G., Kowalczuk M. (2018). Biomass Extraction Using Non-Chlorinated Solvents for Biocompatibility Improvement of Polyhydroxyalkanoates. Polymers.

[15] Rosengart A., Cesário M.T., de Almeida M.C.M.D., Raposo R.S., Espert A., de Apodaca E.D., da Fonseca M.M.R. (2015). Efficient P(3HB) Extraction from Burkholderia Sacchari Cells Using Non-Chlorinated Solvents. Biochem. Eng. J..

[16] López-Abelairas M., García-Torreiro M., Lú-Chau T., Lema J.M., Steinbüchel A. (2015). Comparison of Several Methods for the Separation of Poly(3-Hydroxybutyrate) from Cupriavidus Necator H16 Cultures. Biochem. Eng. J..

[17] Jiang Y., Mikova G., Kleerebezem R., van der Wielen L.A., Cuellar M.C. (2015). Feasibility Study of an Alkaline-Based Chemical Treatment for the Purification of Polyhydroxybutyrate Produced by a Mixed Enriched Culture. AMB Express.

[18] Pospisilova A., Novackova I., Prikryl R. (2021). Isolation of Poly(3-Hydroxybutyrate) from Bacterial Biomass Using Soap Made of Waste Cooking Oil. Bioresour. Technol..

[19] Schindl A., Hagen M.L., Muzammal S., Gunasekera H.A.D., Croft A.K. (2019). Proteins in Ionic Liquids: Reactions, Applications, and Futures. Front. Chem..

[20] Mezzetta A., Becherini S., Pretti C., Monni G., Casu V., Chiappe C., Guazzelli L. (2019). Insights into the Levulinate-Based Ionic Liquid Class: Synthesis, Cellulose Dissolution Evaluation and Ecotoxicity Assessment. New J. Chem..

[21] Zheng S., Nie Y., Zhang S., Zhang X., Wang L. (2015). Highly Efficient Dissolution of Wool Keratin by Dimethylphosphate Ionic Liquids. ACS Sustain. Chem. Eng..

[22] Hecht S.E., Niehoff R.L., Narasimhan K., Neal C.W., Forshey P.A., Phan D.V., Brooker A.D.M., Combs K.H. (2010). Extracting Biopolymers from a Biomass Using Ionic Liquids. U.S. Patent.

[23] Kobayashi D., Fujita K., Nakamura N., Ohno H. (2015). A Simple Recovery Process for Biodegradable Plastics Accumulated in Cyanobacteria Treated with Ionic Liquids. Appl. Microbiol. Biotechnol..

[24] Dubey S., Bharmoria P., Gehlot P.S., Agrawal V., Kumar A., Mishra S. (2018). 1-Ethyl-3-Methylimidazolium Diethylphosphate Based Extraction of Bioplastic “Polyhydroxyalkanoates” from Bacteria: Green and Sustainable Approach. ACS Sustain. Chem. Eng..

[25] Cho C.-W., Pham T.P.T., Zhao Y., Stolte S., Yun Y.-S. (2021). Review of the Toxic Effects of Ionic Liquids. Sci. Total Environ..

[26] Egorova K.S., Gordeev E.G., Ananikov V.P. (2017). Biological Activity of Ionic Liquids and Their Application in Pharmaceutics and Medicine. Chem. Rev..

[27] Carlozzi P., Di Lorenzo T., Ghanotakis D.F., Touloupakis E. (2020). Effects of PH, Temperature and Salinity on P3HB Synthesis Culturing the Marine Rhodovulum Sulfidophilum DSM-1374. Appl. Microbiol. Biotechnol..

[28] Padovani G., Emiliani G., Giovanelli A., Traversi M.L., Carlozzi P. (2018). Assessment of Glycerol Usage by Five Different Purple Non-Sulfur Bacterial Strains for Bioplastic Production. J. Environ. Chem. Eng..

[29] Carlozzi P., Touloupakis E. (2021). Bioplastic Production by Feeding the Marine Rhodovulum Sulfidophilum DSM-1374 with Four Different Carbon Sources under Batch, Fed-Batch and Semi-Continuous Growth Regimes. New Biotechnol..

[30] Carlozzi P., Touloupakis E., Di Lorenzo T., Giovannelli A., Seggiani M., Cinelli P., Lazzeri A. (2019). Whey and Molasses as Inexpensive Raw Materials for Parallel Production of Biohydrogen and Polyesters via a Two-Stage Bioprocess: New Routes towards a Circular Bioeconomy. J. Biotechnol..

[31] Carlozzi P., Giovannelli A., Traversi M.L., Touloupakis E. (2021). Poly(3-Hydroxybutyrate) Bioproduction in a Two-Step Sequential Process Using Wastewater. J. Water Process. Eng..

[32] Carlozzi P., Giovannelli A., Traversi M.L., Touloupakis E., Di Lorenzo T. (2019). Poly-3-Hydroxybutyrate and H2 Production by Rhodopseudomonas Sp. S16-VOGS3 Grown in a New Generation Photobioreactor under Single or Combined Nutrient Deficiency. Int. J. Biol. Macromol..

[33] Guglielmero L., Mezzetta A., Pomelli C.S., Chiappe C., Guazzelli L. (2019). Evaluation of the Effect of the Dicationic Ionic Liquid Structure on the Cycloaddition of CO2 to Epoxides. J. CO2 Util..

[34] Chiappe C., Margari P., Mezzetta A., Pomelli C.S., Koutsoumpos S., Papamichael M., Giannios P., Moutzouris K. (2017). Temperature Effects on the Viscosity and the Wavelength-Dependent Refractive Index of Imidazolium-Based Ionic Liquids with a Phosphorus-Containing Anion. Phys. Chem. Chem. Phys..

[35] Ramsay J.A., Berger E., Voyer R., Chavarie C., Ramsay B.A. (1994). Extraction of Poly-3-Hydroxybutyrate Using Chlorinated Solvents. Biotechnol. Tech..

[36] Rosengart A. (2013). A Study to Improve the Extraction Yields of Poly 3-Hydroxybutyrate from Burkholderia Sacchari Cells Avoiding Chlorinated Solvents.

[37] Ghani N.A., Sairi N.A., Aroua M.K., Alias Y., Yusoff R. (2014). Density, Surface Tension, and Viscosity of Ionic Liquids (1-Ethyl-3-Methylimidazolium Diethylphosphate and 1,3-Dimethylimidazolium Dimethylphosphate) Aqueous Ternary Mixtures with MDEA. J. Chem. Eng. Data.

[38] Zhang Z., Zhang X., Nie Y., Wang H., Zheng S., Zhang S. (2017). Effects of Water Content on the Dissolution Behavior of Wool Keratin Using 1-Ethyl-3-Methylimidazolium Dimethylphosphate. Sci. China Chem..

[39] Chen X., Liang S., Wang S.-W., Colby R.H. (2018). Linear Viscoelastic Response and Steady Shear Viscosity of Native Cellulose in 1-Ethyl-3-Methylimidazolium Methylphosphonate. J. Rheol..

[40] Jurasek L., Marchessault R.H. (2004). Polyhydroxyalkanoate (PHA) Granule Formation in Ralstonia Eutropha Cells: A Computer Simulation. Appl. Microbiol. Biotechnol..

[41] Golomysova A., Gomelsky M., Ivanov P.S. (2010). Flux Balance Analysis of Photoheterotrophic Growth of Purple Nonsulfur Bacteria Relevant to Biohydrogen Production. Int. J. Hydrogen Energy.

[42] McKinlay J.B., Harwood C.S. (2011). Calvin Cycle Flux, Pathway Constraints, and Substrate Oxidation State Together Determine the H_2_ Biofuel Yield in Photoheterotrophic Bacteria. MBio.

[43] McCully A.L., McKinlay J.B. (2016). Disrupting Calvin Cycle Phosphoribulokinase Activity in Rhodopseudomonas Palustris Increases the H2 Yield and Specific Production Rate Proportionately. Int. J. Hydrogen Energy.

[44] Carlozzi P., Seggiani M., Cinelli P., Mallegni N., Lazzeri A. (2018). Photofermentative Poly-3-Hydroxybutyrate Production by Rhodopseudomonas Sp. S16-VOGS3 in a Novel Outdoor 70-L Photobioreactor. Sustainability.

[45] Van Walsem J., Zhong L., Shih S.S. (2007). Polymer Extraction Methods 2010. U.S. Patent.

[46] Masood F., Hasan F., Ahmed S., Hameed A. (2012). Biosynthesis and Characterization of Poly(3-Hydroxybutyrate-Co-3-Hydroxyvalerate) from Bacillus Cereus FA11 Isolated from TNT-Contaminated Soil. Ann. Microbiol..

[47] Bujok J., Gąsior-Głogowska M., Marszałek M., Trochanowska-Pauk N., Zigo F., Pavľak A., Komorowska M., Walski T. (2019). Applicability of FTIR-ATR Method to Measure Carbonyls in Blood Plasma after Physical and Mental Stress. BioMed Res. Int..

[48] Gunaratne L.M.W.K., Shanks R.A. (2005). Multiple Melting Behaviour of Poly(3-Hydroxybutyrate-Co-Hydroxyvalerate) Using Step-Scan DSC. Eur. Polym. J..

